# Premenstrual Syndrome and Depression Among Students Attending Nursing Training Schools in Yangon

**DOI:** 10.1192/j.eurpsy.2026.11923

**Published:** 2026-07-31

**Authors:** E. T. Z. Thwe

**Affiliations:** 1 Department of Psychiatry, Interim University of Medicine 1, Yangon, Myanmar; 2 Lancashire and South Cumbria NHS Foundation Trust, Blackburn, United Kingdom

## Abstract

**Introduction:**

Premenstrual Syndrome (PMS) is a common problem among women of reproductive age and is associated with impairment in physical, psychological, and social functioning. Depression is one of common disorders that may co-occur with PMS, and the two conditions can negatively influence the severity of each other. Recognising the co-occurrence of these two disorders is important for timely and effective management. Although some local research has addressed women’s mental health problems during perinatal and perimenopausal periods, there remains limited evidence on PMS and its psychiatric comorbidities in Myanmar. This gap suggests that PMS and its associated mental health challenges are often overlooked in local research.

**Objectives:**

This study aimed to describe the prevalence of PMS and depression and to examine their association among nursing students in Yangon.

**Methods:**

This study employed a cross-sectional descriptive design. The study population included female students from Nursing Training Schools in Yangon. Students who were pregnant or taking any type of hormonal contraception were excluded. PMS symptoms were assessed by using the Premenstrual Symptoms Screening Tool (PSST), a validated, user-friendly instrument that identifies clinically significant PMS and Premenstrual Dysphoric Disorder (PMDD) according to DSM-IV criteria. Depressive symptoms were assessed using the Beck Depression Inventory II (BDI II). Data were analysed using descriptive statistics and Chi-square tests to determine associations.

**Results:**

A total of 90 students participated in the study. Moderate to severe PMS was present in 38.9% of participants, and 12.2% met the criteria for PMDD. Regarding depression, 50% reported no depression, while 28.9% had mild, 17.8% moderate, and 3.3% severe depression. Among students with moderate to severe PMS, 54.3% reported depression compared with 47.3% in the non-PMS group. Although depression was more frequent in the PMS group, the difference was not statistically significant (*p*=0.333). Analysis of background characteristics showed that BMI distribution was significantly associated with PMS (*p*=0.047), with overweight and obesity more common among students with PMS.

**Conclusions:**

A considerable proportion of nursing students experienced moderate to severe PMS, and half of the study population reported some degree of depression. Although PMS was not significantly associated with depression, the high prevalence of both conditions highlights the need for routine screening, mental health support, and lifestyle interventions in this population. The findings of this study underline the importance of addressing PMS among the women population as well as integrating screening practices for PMS symptoms in women presenting with depression (and vice versa).

**Disclosure of Interest:**

None Declared

